# Comparative case study on the implementation of the multi- space concept in a healthcare facility: study protocol

**DOI:** 10.3389/fpsyg.2026.1964644

**Published:** 2026-09-25

**Authors:** Sophia Sachs, Sarah Lütke Lanfer, Claas Lahmann

**Affiliations:** Department of Psychosomatic Medicine and Psychotherapy, Medical Center-University of Freiburg, Faculty of Medicine, University of Freiburg, Freiburg im Breisgau, Germany

**Keywords:** activity-based working (ABW), fatigue, multi-space office, office design, psychosocial work environment, psychosomatic complaints, well-being, workplace health

## Abstract

**Background:**

Modern healthcare organizations face increasing psychosocial and organizational demands driven by digitalization, flexible work models, and rising workload. In large university hospitals such as the Medical Center – University of Freiburg, administrative and clinical staff work in highly complex, interdisciplinary environments where well-being, performance, and patient care are closely linked. The physical office environment plays a crucial role in shaping employees’ well-being, motivation, and health. To meet the high cooperative demands in medical centers, multi-space offices are introduced to support collaboration, flexibility, and efficiency. However, their effects in clinical settings remain unclear. The scarce existing research shows inconsistent findings regarding productivity, satisfaction, privacy, and health outcomes. Thus, there is a need for systematic, empirical studies that investigate how office design influences work processes, well-being, and long-term psychosomatic health outcomes among hospital employees.

**Methods:**

The presented study applies a mixed-methods comparative case design. Two office types are compared: Multi-space offices with activity-based workspaces and traditional closed offices with assigned individual or shared rooms. Participants are employees from different professional groups (e.g., physicians, administrative staff) working in these offices (*N* ≥ 40). Data collection combines (1) an electronic questionnaire, (2) an e-diary with repeated daily assessments, and (3) semi-structured interviews with purposive sampling. Validated instruments assess fatigue, psychosomatic complaints, job satisfaction, commitment, work climate, and social stressors. Quantitative data are analyzed using state-of-the-art exploratory and multivariate methods. Qualitative interview data are analyzed using qualitative content analysis according to Mayring, supported by MAXQDA. Methodological triangulation integrates quantitative and qualitative findings to explore relationships between office design, work processes, and employee well-being.

**Conclusion:**

This study addresses a key gap in healthcare research by systematically examining how multi-space office concepts influence work processes, psychosocial risk and protective factors, as well as employees’ well-being in clinical settings. By linking spatial design with organizational and individual factors, it contributes evidence needed to improve workplace conditions in highly demanding healthcare environments. The use of multiple data sources and analytical strategies allows for the examination of complex relationships between variables. These insights can guide evidence-based decisions on office design and preventive measures to enhance sustainable work environments in healthcare.

**Clinical trial registration:**

https://drks.de/search/de/trial/DRKS00037660/details, identifier DRKS00037660.

## 1 Introduction

Technological advances, digitalization, globalization, demographic change, and, more recently, the COVID-19 pandemic have fundamentally transformed the modern world of work, making the influence of physical work environments on employees an increasingly important topic in occupational and organizational research. Previous research demonstrated that the physical characteristics of office environments can have a significant impact on employees’ well-being, job satisfaction, motivation, work behavior, and health (Al Horr et al., 2016; Bauer et al., 2014; Hoendervanger et al., 2018; Kamarulzaman et al., 2011; Rashid and Zimring, 2008; Veitch et al., 2007). These findings are particularly relevant given that more than half of the German workforce is employed in office-based settings and spends a considerable proportion of their working time in such environments (Hammermann and Stettes, 2023).

To explain these relationships, several theoretical frameworks have been proposed (e.g., Windlinger et al., 2014). Many of these draw on the Job Demands–Resources Model (Bakker and Demerouti, 2007) or the Transactional Stress Model (Lazarus and Launier, 1981). However, as these models were not originally developed to account for the physical work environment, they provide only limited explanations of the interaction between physical workplace characteristics and the psychosocial work environment. Consequently, research on the psychological effects of office environments has increasingly been grounded in Person–Environment (P–E) Fit Theory. This perspective emphasizes the alignment between individuals and their work environments (Caplan, 1987), suggesting that a good fit promotes well-being and satisfaction, whereas a poor fit may contribute to stress (Lütke Lanfer et al., 2021). From this perspective, well-designed office environments seek to enhance person–environment fit by enabling employees to choose workspaces that best match their individual needs. Building on this perspective, the concept of Task–Environment (T–E) Fit focuses more specifically on the correspondence between task requirements and the selected workspace (Bauer et al., 2021). By providing a variety of task-specific work settings, modern office concepts aim to improve productivity by better aligning work activities with appropriate spatial conditions (Bauer et al., 2021; De Paoli et al., 2013).

One office concept that explicitly follows this principle is the multi-space-office. Multi-space offices have emerged as an evolution of the traditional open-plan office with the aim to optimize the interaction between individuals and their work environments and to better address the challenges associated with increasing complexity, information overload, and pressure to innovate. They combine open and enclosed work areas with additional functional spaces, including meeting rooms, quiet work areas, telephone booths, break and retreat rooms, and conference facilities (Becker et al., 2020; Wohlers and Hertel, 2017). These multifunctional environments are designed to support activity-based working (ABW) by matching specific spatial configurations to different work activities (Becker et al., 2019; Seiferlein and Kohlert, 2018). Employees can therefore flexibly choose and switch between workspaces according to their task requirements (Gerdenitsch and Korunka, 2018). This flexibility is often enabled through desk-sharing arrangements, in which workstations are not permanently assigned to individual employees. Instead, desks are used according to availability and current work demands - a practice commonly referred to as flexible office systems or “hot desking” (Bauer et al., 2021).

Given these potential advantages, multi-space offices have become the predominant outcome of new construction, renovation, and office redesign projects (Kratzer, 2017). Ongoing transformations in the modern world of work have further accelerated the development and implementation of flexible office concepts (Becker et al., 2019; Lütke Lanfer et al., 2021). Reflecting this practical relevance, the design of multi-space environments has received increasing attention in both research and practice over recent years. Nevertheless, important research gaps remain. While post-pandemic research has primarily focused on organizational outcomes such as productivity, collaboration, and innovation (e.g., Bäcklander and Richter, 2022; Candido et al., 2021; Forooraghi et al., 2023), comparatively less attention has been paid to employee-related outcomes. Another research gap remains regarding the implementation of multi-space offices in specific work contexts. In particular, empirical evidence on the application of multi-space offices in clinical and medical settings is scarce, as existing research has predominantly focused on conventional office environments across various industries (Candido et al., 2021). Given the specific demands of healthcare environments, including high cognitive demands, confidentiality requirements, and the need for effective communication and collaboration, further research is needed to understand whether findings from general office settings can be transferred to medical contexts. Moreover, findings regarding the effects of specific features of multi-space offices remain heterogeneous and, in some cases, contradictory (e.g., Marzban et al., 2023). Against this background, the following section summarizes the current evidence on the effects of multi-space offices on employees, highlighting both consistent findings and existing inconsistencies as well as identifying important directions for future research.

With respect to social relationships, communication, and collaboration, several studies report positive effects, including enhanced collaboration, increased interaction, and a stronger sense of community (Arundell et al., 2018; Budie et al., 2019; Divvet, 2020; Gerdenitsch et al., 2017; Haner and Wackernagel, 2018; Mache et al., 2020). In contrast, other studies indicate reduced satisfaction with communication and a decline in face-to-face interactions, potentially due to reduced accessibility or a shift toward digital communication channels (Bernstein and Turban, 2018; De Been and Beijer, 2014; Haapakangas et al., 2019; Muhonen and Berthelsen, 2020). Still other studies report mixed or negligible effects (De Been et al., 2015; Rolfö et al., 2018a; Wohlers and Hertel, 2018).

Regarding privacy, concentration, interruptions, and distractions, acoustic and visual disturbances are frequently identified as major drawbacks of multi-space offices (Barnes et al., 2020; Bauer et al., 2021). These factors may impair concentration, reduce cognitive performance, and compromise perceived privacy (De Been and Beijer, 2014; Bodin Danielsson and Bodin, 2008; Haapakangas et al., 2018). Providing sufficient quiet areas appears to mitigate some of these negative effects (Bauer et al., 2021). Although interruptions can have both beneficial and detrimental consequences, they are often perceived negatively by employees (Lütke Lanfer et al., 2021). Their impact depends on multiple factors, including task characteristics, individual differences, and the medium of interruption, and may range from increased stress, tension, and time delays to enhanced creativity, innovation, and information exchange (Baethge and Rigotti, 2013; Becker et al., 2021; Foroughi et al., 2016; Eliav and Miron-Spektor, 2015; Sio and Ormerod, 2009; Okhuysen and Eisenhardt, 2002). For tasks requiring high levels of concentration, employees generally prefer acoustically and visually shielded environments with minimal potential for disruption (Bauer et al., 2021). Positive outcomes in this domain appear to be associated with low occupancy density and the availability of sufficient alternative workspaces (Hoendervanger et al., 2018, 2022; Rolfö et al., 2018b).

Findings on productivity are similarly inconsistent (Becker et al., 2019; Masoudinejad and Veitch, 2023). Some studies report positive associations between frequent workspace changes, higher satisfaction, and increased productivity (Candido et al., 2019; Divvet, 2020; Hoendervanger et al., 2016; Shinoyama et al., 2018). Others highlight negative effects, particularly due to distractions and the time required to locate suitable workspaces (Arundell et al., 2018; De Been and Beijer, 2014; Haapakangas et al., 2019; Herbig et al., 2016; Pitchforth et al., 2020; Öhrn et al., 2021). Again, some studies report mixed or null findings (Haapakangas et al., 2018; Haynes et al., 2019; Jahncke and Hallman, 2020; Rolfö et al., 2018a; Wadu Mesthrige and Chiang, 2019).

Evidence regarding job satisfaction and organizational commitment is equally inconclusive (Bauer et al., 2021). Positive associations have been reported, often linked to improved perceptions of architecture, layout, aesthetics, and interior design (Arundell et al., 2018; Becker et al., 2019, 2020; Brunia et al., 2016; Candido et al., 2019; De Been and Beijer, 2014; De Been et al., 2015; De Leede and Nijland, 2016; Ekstrand and Damman, 2016; Halldorsson et al., 2022; Haner and Wackernagel, 2018; Lusa et al., 2019; Rolfö et al., 2018b; Shinoyama et al., 2019). At the same time, negative effects -such as unfavorable climatic conditions and reduced overall satisfaction - have also been documented (Becker et al., 2019; Herbig et al., 2016; Pitchforth et al., 2020). In some cases, both positive and negative outcomes have been observed within the same samples (Haapakangas et al., 2018; Haynes et al., 2019; Rolfö et al., 2018a; Wadu Mesthrige and Chiang, 2019). Overall, there is no clear consensus on whether increased autonomy, altered cognitive demands, and improved indoor environmental quality translate into enhanced satisfaction.

A similarly inconclusive picture emerges with respect to employee health. Systematic reviews examining the relationship between office design and health (e.g., De Croon et al., 2005; Engelen et al., 2019; Ford et al., 2022; Marzban et al., 2023) highlight both the limited number of studies and the heterogeneity of findings. Evidence regarding general health outcomes, short-term psychological effects (e.g., exhaustion, need for recovery), and long-term psychosomatic consequences remains inconsistent. While some studies report dissatisfaction with ergonomic conditions and insufficient equipment (Babapour Chafi et al., 2020), others indicate potential benefits, such as increased physical activity and reduced sedentary behavior (Arundell et al., 2018; Jindo et al., 2019; Sugiyama et al., 2021; Wahlström et al., 2019). Similarly, findings on perceived health are mixed, and the effects on mental well-being and psychosomatic symptoms remain unclear (Becker et al., 2020). Notably, one meta-analysis suggests an increased risk of short-term sickness absence in flexible and open-plan office environments (Mauss et al., 2023). These inconsistencies are also reflected in recent systematic reviews (e.g., Marzban et al., 2023).

Taken together, the literature provides no clear consensus regarding the effects of multi-space offices on employee outcomes. While flexible office concepts may enhance collaboration, workspace fit, and certain aspects of satisfaction, they may simultaneously impair privacy, concentration, and health depending on contextual and organizational conditions. These inconsistencies suggest that the effectiveness of multi-space-offices is unlikely to depend solely on the physical office layout but rather on its implementation, use, and contextual moderators (Ansio et al., 2020; Lütke Lanfer and Pauls, 2017; Marzban et al., 2023). Accordingly, research has increasingly shifted from asking whether multi-space offices are effective to examining the conditions under which they promote positive employee outcomes. These influencing factors can be broadly categorized into spatial design, organizational conditions, and individual characteristics.

From a perspective of spatial design characteristics, key factors include zone diversity, spatial separation, functionality of furniture and tools, technological integration, ergonomics, personalization options, as well as aesthetics and acoustics (Babapour et al., 2018; Bauer et al., 2021; Becker et al., 2022; Haner and Wackernagel, 2018; Kratzer, 2020; Marquardt et al., 2002). Poor acoustics, insufficient ergonomics, and limited opportunities for personalization have been identified as risk factors for employees, whereas high spatial diversity, strong task–environment fit, and modern, attractive equipment appear to have beneficial effects (Becker et al., 2019, 2021, 2022).

However, the physical environment alone does not determine employee outcomes. Organizational conditions also play a crucial role in shaping how multi-space offices are implemented and experienced. A supportive organizational culture and leadership style are essential for successful implementation (Haner and Wackernagel, 2018). In line with change management theories, research highlights the importance of management support during the transition to multi-space environments and its impact on user satisfaction (Babapour, 2019; Brunia et al., 2016; Ianeva et al., 2015; Rolfö, 2018; Wahlström et al., 2019). Aligning ABW concepts with organizational culture and involving employees in decision-making processes before and after implementation are associated with higher satisfaction over time (Babapour, 2019; Christersson et al., 2017). Additionally, clear rules and norms governing shared workspace use are critical (Ansio et al., 2020; Babapour et al., 2018; Budie et al., 2019; Göçer et al., 2018). Greater autonomy and control over work conditions - including workload, scheduling, social interactions, and environmental factors - have been linked to improved mental health, well-being, and performance (Fincke et al., 2020), as well as increased resilience (Becker et al., 2020; Lütke Lanfer and Becker, 2020).

Even under identical spatial and organizational conditions, employees differ in how they experience multi-space offices. Individual characteristics therefore represent a third important category of influencing factors. Personality traits, age, and gender appear to moderate these effects. For example, introverted individuals tend to prefer more private workspaces, while extroverted individuals likewise report reduced productivity under conditions of limited privacy (Wadu Mesthrige and Chiang, 2019). Communication patterns have been found to correlate positively with extraversion and negatively with age (Budie et al., 2019). Evidence regarding gender differences is mixed, although some findings suggest greater sensitivity among female employees to environmental conditions such as clean desk policies (Göçer et al., 2018). Additional factors include work-related behaviors such as task–environment crafting, daily planning, and frequency of workspace changes, with more frequent switching associated with higher satisfaction (Bäcklander et al., 2021; Häne and Windlinger, 2022; Hoendervanger et al., 2016). Other potentially relevant variables—such as social support, sense of belonging, organizational culture, self-efficacy, and work roles—have been discussed but remain underexplored empirically.

In summary, the existing literature indicates that multi-space offices can have both beneficial and adverse effects on employee productivity, well-being and health. While they may enhance autonomy, social interaction, and resilience, they also pose risks related to cognitive load, interruptions, and stress (Becker et al., 2019; Haner and Wackernagel, 2018; Lütke Lanfer et al., 2021). Furthermore, the findings suggest that the effects of multi-space offices cannot be attributed to the physical environment alone but emerge from the interaction between spatial design, organizational implementation, and individual employee characteristics. Understanding these interactions may help explain the heterogeneous findings reported in the existing literature and guide future research toward identifying the conditions under which multi-space offices are most effective. To support the development of preventive and health-promoting workplace environments, it is therefore essential to identify the contextual factors that shape employees’ experiences and determine under which conditions multi-space offices contribute to positive outcomes.

Therefore, the present study investigates the effects of a multi-space office concept compared with traditional office settings in a medical facility. To guide this investigation, a conceptual framework was developed based on the existing literature, integrating spatial characteristics, work processes, individual and organizational protective and risk factors, and short- and long-term indicators of employee well-being. The framework (Figure 1) provides the theoretical basis for examining how different office environments may shape employee experiences and outcomes within a clinical context.

**FIGURE 1:**
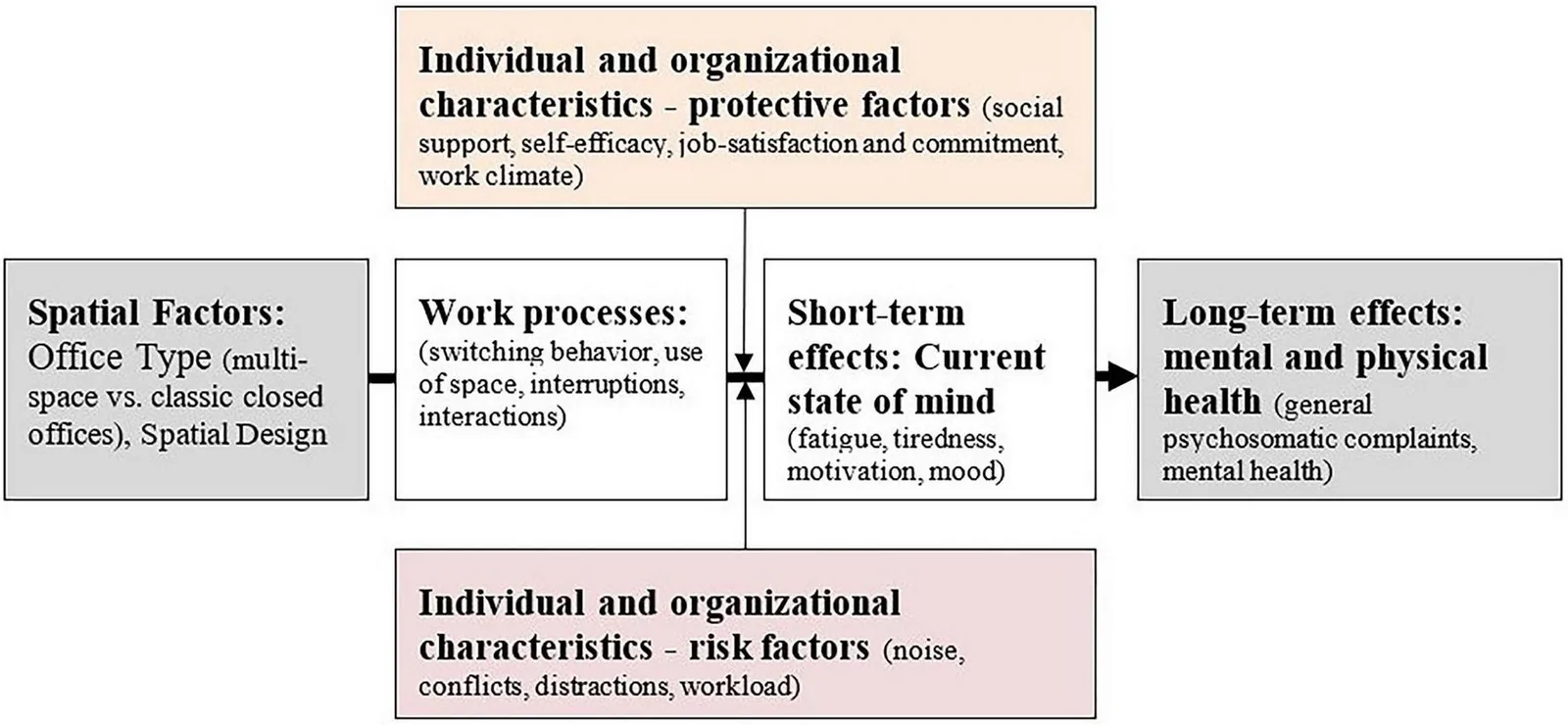
Conceptual framework-schematic overview of central variables.

## 2 Methods and analysis

### 2.1 Aim

Building on this conceptual framework (Figure 1), the primary aim of this exploratory study is to examine the relationships between office design, work processes, protective and risk factors, and both short- and long-term outcomes related to employee well-being in a clinical setting. Specifically, this study examines how spatial, organizational, and individual factors interact in different office environments to influence employees’ mental and physical well-being and seeks to identify structural, social, and spatial starting points for preventive improvements in employee health. Ultimately, the study aims to provide empirical evidence on the benefits and risks of modern office concepts in healthcare and to derive practical recommendations for designing healthy, efficient, and sustainable work environments in hospitals.

### 2.2 Design and setting

To achieve these objectives, the study adopts an exploratory mixed-methods design using a questionnaire survey, an electronic diary study, and semi-structured interviews to compare clinical teams that are working in multi-space offices and in traditional, closed office settings.

The study is conducted in three specialist clinics at a German university children’s hospital, all of which relocated to the same newly constructed building in September 2024. This transition involved not only organizational restructuring but also the implementation of different office concepts, with some teams relocating in traditional offices while others transitioned to multi-space environments. Thus, office type was determined by the respective organizational and spatial arrangements of the clinics and was not assigned by the researchers. Accordingly, the study is designed as an exploratory observational case study to compare different office environments rather than as a causal evaluation of the effects of office design. The study is monocentric, observational in nature, and does not involve any intervention, focusing solely on diagnostic assessment.

Data collection will take place approximately 15 months after the relocation. Thus, the study does not assess the immediate transition period or constitute a pre-post evaluation of the relocation. Instead, it examines employees’ experiences of the established office environments after an extended period of adaptation to the new building and office concepts.

### 2.3 Study population

Given the exploratory mixed-methods design, the study does not aim to test predefined hypotheses but to explore complex relationships between office environment, work processes, and employee well-being. Accordingly, a formal power calculation for hypothesis testing was not used to determine the minimum sample size. Considering the clinical setting and the integration of qualitative methods, a pragmatic minimum sample size of *N* = 40 participants (approximately 20 per group) was defined. This minimum target was chosen to allow descriptive analyses, exploratory comparisons, and the integration of quantitative and qualitative findings. *N* = 40 was considered a minimum rather than a fixed sample size, and recruitment efforts are made to include as many eligible participants as possible beyond this minimum. The final sample size will determine the feasibility and complexity of the planned exploratory quantitative analyses.

Inclusion criteria are: (1) legal age, (2) German language proficiency at B2 level, (3) employment at the clinic for at least 1 month and (4) on-site presence of at least 3 days per week. Individuals who do not meet these criteria will be excluded. Efforts will be made to ensure comparability between teams, except for office type.

Participants will be recruited via internal communication channels. Study information will be distributed digitally by clinic management, including details on purpose, procedures, and data protection. Additional channels (e.g., intranet announcements, QR codes) may be used to increase reach and transparency. Potential participants can access the questionnaire survey directly via a link, ensuring that participation is self-initiated and on a voluntary basis. All professional groups from the three clinics are invited to participate. This includes medical staff, scientific employees, pharmacists, nurses, administrative staff, and educators who work physically close together. The profession is also recorded as a covariate.

All participants completing the questionnaire will be invited to take part in the diary study. Automated reminders are used to support timely completion of diary entries. Interview participants will be selected from survey respondents who indicate willingness to participate, aiming to achieve variation across professional groups and office types. The number of semi-structured interviews will be determined iteratively based on participant availability, willingness to participate, and achievement of data saturation, with approximately 10–15 interviews anticipated.

### 2.4 Study procedure

The study is conducted in three phases (Figure 2). During the first phase, clinical teams are recruited, researchers are trained, the interview guide is developed, and the electronic diary study is pilot-tested.

**FIGURE 2:**
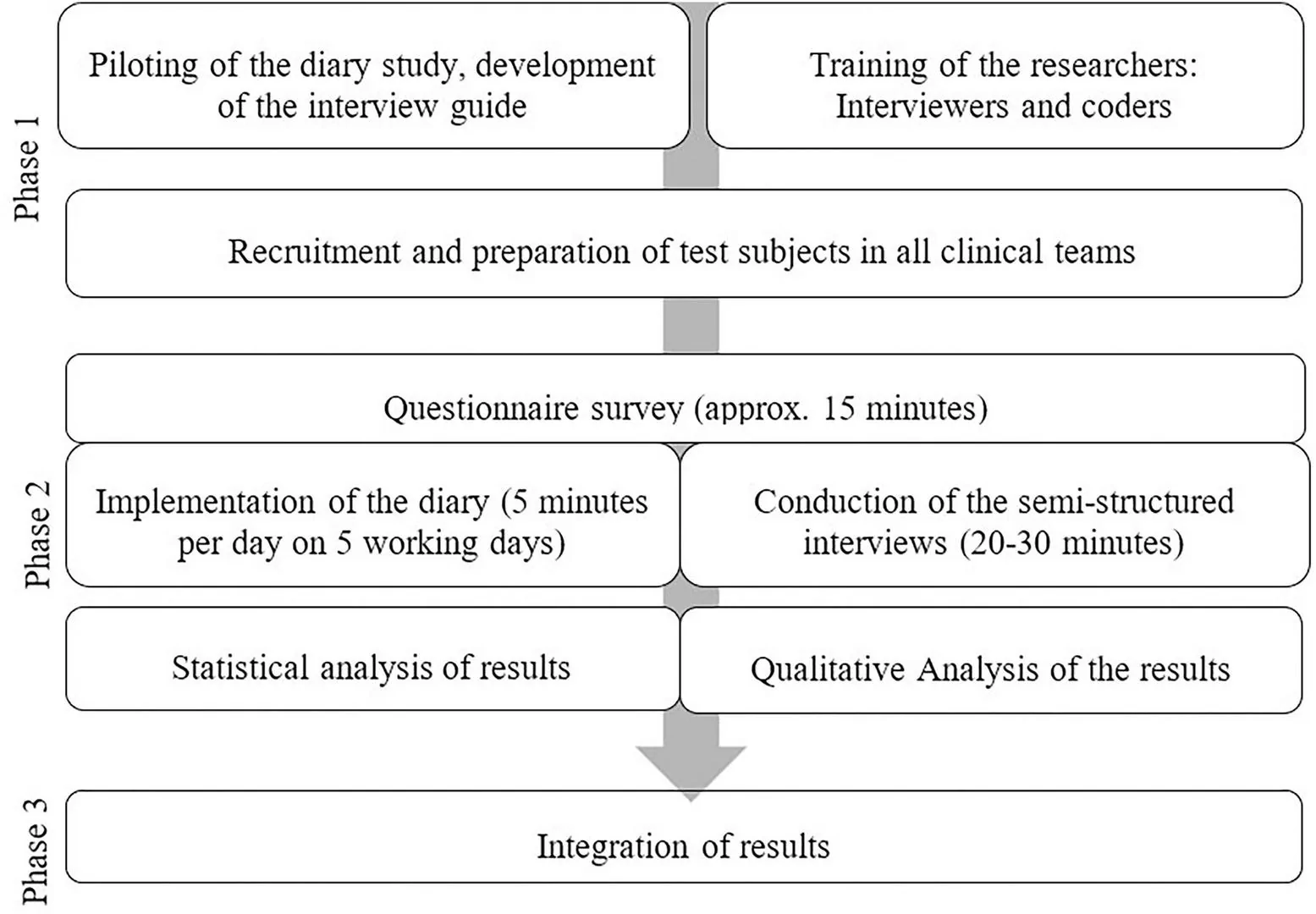
Study procedure.

The second phase comprises data collection. At the beginning, participants receive electronic access to the questionnaire, including detailed information about the study procedures, potential risks, and contact persons. Informed consent to participate in the study and to the processing of their data for research purposes is obtained electronically prior to participation. Participants may withdraw from the study at any time without providing a reason. Once the participants have completed the questionnaire survey, the diary survey is distributed. At the same time, the interviews are expected to be conducted over a span of 3 weeks. The total time commitment per participant is estimated at 60–90 min.

The third phase consists of data analysis, integration of quantitative and qualitative findings, and preparation of the final report. The results will be communicated to the participating clinics in aggregated form. The total study duration for individual participants is approximately 4 months, from initial participation to dissemination of study findings.

### 2.5 Material and outcomes

As previously stated, data collection comprises a questionnaire survey, an electronic diary study, and semi-structured interviews.

The questionnaire survey assesses demographic characteristics, relevant control variables, and study outcomes. Demographic information includes age, gender, occupational group, professional experience, length of employment, and previous experience with different office concepts. Control variables include job demands, shift work, patient contact, access to home office, desk-sharing experience, and workspace satisfaction. In addition, established instruments are used to assess relevant psychological and organizational constructs, including fatigue, psychosomatic complaints, self-efficacy, job satisfaction, organizational commitment, work-related quality of life, working climate, and workplace conflicts (Table 1). The selection of measures was guided by theoretical relevance, psychometric quality, and feasibility within the clinical setting. The questionnaire is designed to require approximately 15–20 min to complete. The number of items was intentionally limited to reduce participant burden while maintaining adequate coverage of the relevant constructs. Data are collected and managed electronically using REDCap (Research Electronic Data Capture), hosted at University Medical Center Freiburg (Harris et al., 2009).

**TABLE 1:** Overview of variables.

Variable	Operationalization
Questionnaire	
Demographic variables	Age, gender, occupational group, duration and type of employment, previous experience with office concepts (multi-space previous experience yes/no), 8 items
Control variables	Weekly working hours, shift work (yes/no, which shifts), contact with patients (yes/no, frequency), home office access, desk sharing, 5 items
Fatigue	FACIT Fatigue Scale (Montan et al., 2018), 13 items
General psychosomatic complaints	Psychosomatic complaints in a non-clinical context (Mohr and Müller, 2014), 20 items Short questionnaire for psychological and physical complaints SCL-K-9 (Petrowski et al., 2019), 9 items
Self-efficacy	General Self-Efficacy Short Scale (ASKU) (Beierlein et al., 2014), 3 items
Personality	Big Five Inventory (BFI-10) (Rammstedt et al., 2014), 10 items
Job satisfaction and commitment	Short questionnaire for recording general and facet-specific job satisfaction, KAFA (Haarhaus, 2015), 6 items Organizational Commitment Questionnaire (OCQ-G, Maier and Woschée, 2014), 15 items
Work climate	Subjective satisfaction and stress of work and occupation, subscale Work climate and work and occupational stress (Weyer et al., 2014), 20 items
Current conflicts	Social stressors at the workplace K, (Frese and Zapf, 1987), 10 items
E-Diary	
Noise	“Do you currently perceive disturbing noises in your working environment?” Yes/no If yes: “How loud do you currently perceive the noise?” (1 = very quiet, 5 = very loud) “How much does the noise affect you at work?” (1 = not at all, 5 = very much) 3 items
Use of space/change of workplace	“How satisfied are you with your current workplace?” 1 = not at all, 5 = very satisfied “Have you already changed your workplace today?” → Yes/No If Yes: “How often have you changed workplaces so far?” → Open number “How much have you moved around today (e.g., walking, standing)”? 1 = not at all, 5 = very much 4 items
Work process	“How often were you interrupted by external circumstances today?” → 1 = never, 5 = very often “How heavy is your current workload?” → 1 = very low, 5 = very high “How satisfied are you with your current work performance?” → 1 = not at all, 5 = very satisfied 3 items
Current state	Items based on the Multidimensional Mood Questionnaire (MDBF, Steyer et al., 2004, short version) - “I am currently feeling…” → “tired - awake” “I am currently…” → “listless - motivated” “I am currently…” → “in a bad mood - in a good mood” 3 items
Social variables	Involvement and social support (modified from Berlin Social Support Scales, Schulz and Schwarzer, 2003): 1 = not at all, 5 = very strongly “I feel involved in my team right now” “I experienced support from colleagues today” “I exchanged ideas with colleagues today”. “I had the feeling that I was doing something meaningful today”. 4 items

The electronic diary study is also implemented using REDCap. Over a 3-week observation period, participants complete diary entries on at least five consecutive working days. Days on which participants work from home or at another workplace outside the clinic are excluded. Three prompts are delivered per day: two at fixed times (10:00 and 16:00) and one at a randomly selected time to capture variability in daily experiences. Participants are required to complete each entry within 30 min of receiving the prompt; unanswered prompts are recorded as missing. Each diary entry requires approximately 5 min to complete. Participants are included in the diary analyses if they complete at least 60% of the scheduled assessments. Single-item measures assess perceived noise exposure, workspace use, work processes, current well-being, and social interactions (Table 1).

Semi-structured interviews are conducted to complement the quantitative data and last approximately 20–30 min. An interview guide was developed based on the conceptual framework, reviewed by three experts, and pilot-tested prior to use. Topics include workspace use and perceived benefits, changes in work processes following relocation, social dynamics (including conflicts, relationships, interactions, and formal and informal communication), experienced challenges, and coping strategies. For participants working in traditional office environments, the interview guide focuses on experiences related to relocation and changes in the workplace environment rather than changes in office concept.

### 2.6 Data analysis and measures

The quantitative data from the questionnaires and diary study will be recorded electronically and evaluated using an exploratory analysis approach. The analyses aim to describe patterns, examine associations between variables, and identify potential factors related to employee outcomes. The conceptual framework presented in Figure 1 guided the selection and grouping of variables for the exploratory analyses. Variables were categorized into spatial factors, work processes, protective and risk factors, and outcome variables. Figure 1 is intended as a conceptual illustration of the study scope and does not constitute a predefined statistical model.

Descriptive statistics (e.g., means, standard deviations, frequencies, and distributions) will be calculated. Exploratory comparisons between employees working in multi-space offices and traditional office settings will be conducted using appropriate statistical tests depending on data characteristics. Exploratory regression models will be applied to examine associations between office type, relevant covariates (e.g., clinic, occupational group, shift work, and workspace satisfaction), and indicators of employee well-being. Due to the exploratory design and expected sample size, results will primarily be interpreted based on effect estimates and patterns of association rather than confirmatory hypothesis testing.

Missing values are to be expected, especially in the e-diary data. Potential systematic differences between participants with and without missing values will be examined. Depending on the extent and structure of missingness, appropriate methods will be applied, including linear mixed-effects models for repeated diary measurements and, where appropriate, multiple imputation procedures.

To further examine the relationships proposed in the conceptual framework (Figure 1), an exploratory structural equation model (SEM) will be conducted using IBM SPSS Amos, provided that the final sample size and data structure allow for sufficiently stable model estimation. Given the exploratory nature of the study and the anticipated sample size, SEM is not intended as a confirmatory test of predefined hypotheses. Rather, it will be used to explore the plausibility and structure of the hypothesized relationships between spatial characteristics, work processes, individual and organizational protective and risk factors, and employee well-being outcomes. Model parameters, path coefficients, and explained variance will be examined descriptively and interpreted cautiously, with particular consideration of model complexity and sample size. If the available data do not support sufficiently stable estimation of the full model, less complex structural models or alternative multivariate analyses will be considered.

If the final sample size and data structure permit sufficiently stable estimation, an exploratory network analysis may additionally be considered to investigate potential data-driven interrelationships among selected variables from the questionnaire and aggregated diary measures. Given the anticipated sample size, such analyses will not be considered a primary analytical approach. If conducted, regularized network models will be used to reduce the risk of spurious associations, and network characteristics will be interpreted cautiously with regard to their stability and uncertainty. Centrality measures, such as strength, betweenness, and closeness, may be examined descriptively where appropriate. Network visualization may be performed using the Fruchterman–Reingold layout (Jünger and Gärtner, 2023).

Qualitative interview data will be audio-recorded with participants’ consent, transcribed using MAXQDA24 (VERBI Software, 2021), and analyzed following qualitative content analysis as proposed by Mayring (2016). A category system will be developed based on the interview guide and extended through inductive coding. The coding framework will be iteratively refined and validated through consensus among researchers. Text segments will be condensed and analyzed across categories to identify recurring themes, differences, and contextual factors.

To enhance construct validity and provide a comprehensive understanding of the phenomenon under investigation, findings from questionnaires, diary data, and interviews will be integrated through data triangulation. Quantitative patterns will be compared with qualitative themes to identify converging findings, contextual explanations, and potential mechanisms underlying employee experiences in different office environments.

### 2.7 Data management and data protection

This study involves the collection of personal data, including audio recordings of interviews, questionnaire and diary data, informed consent forms, and interview transcripts. Sensitive information, particularly related to health and personal experiences, is collected as part of the survey and diary components. Audio recordings will be stored in a password-protected format, with direct identifiers such as names and institutional affiliations removed. Full anonymization cannot be guaranteed due to the identifiable nature of voice recordings and contextual interview content. Transcripts and quantitative data will therefore be pseudonymized using an externally maintained ID list, ensuring that data can only be linked to participants via secure procedures. All data will be stored on secure servers of the University Hospital in compliance with the General Data Protection Regulation (GDPR) and according to the data safety procedures of the University Medical Center Freiburg. Participation is voluntary, and consent for data processing may be withdrawn at any time without consequences. In the event of withdrawal, no further data will be collected.

## 3 Discussion

This study addresses an important research gap by investigating how multi-space office concepts influence employee well-being and work processes in a clinical healthcare setting. While previous research has examined modern office concepts primarily in general office environments, evidence from healthcare settings remains limited. Existing studies have often focused on isolated aspects of workplace design, such as satisfaction, privacy, or environmental conditions, without considering the complex interplay between spatial characteristics, work processes, individual resources, and organizational factors. The present study therefore aims to provide a more comprehensive understanding of how different office environments shape employee experiences by examining the interactions between spatial, organizational, and individual factors and their associations with short- and long-term indicators of employee well-being. In addition, the study seeks to identify structural, social, and spatial starting points for preventive workplace improvements and to derive practice-oriented recommendations for healthy and sustainable office design in healthcare. A key contribution of this study is the integration of environmental, organizational, and individual perspectives within a single analytical framework, allowing a more comprehensive examination of how office environments interact with work processes and employee well-being.

A key strength of the study is its mixed-methods design, integrating questionnaire data, electronic diary assessments, and qualitative interviews. This approach enables the investigation of both general patterns and employees’ contextualized experiences of workplace environments. The combination of cross-sectional, repeated-measure, and qualitative data provides complementary perspectives on how office concepts may influence work processes and employee well-being. In addition, the study is informed by established theoretical perspectives on work-related stress and resources, including the Job Demands–Resources model and the transactional stress model, which support the interpretation of potential relationships between workplace characteristics and employee outcomes.

However, several limitations should be considered. Given the exploratory nature of the study, the sample size is not intended to provide sufficient statistical power for confirmatory hypothesis testing. Consequently, the precision and stability of quantitative estimates, particularly for more complex multivariate analyses, may be limited. Any such analyses will therefore be interpreted cautiously and primarily as hypothesis-generating. This limitation is addressed through the integration of multiple data sources and the complementary use of qualitative methods to provide contextual interpretation of quantitative findings.

As all three clinics relocated to the same newly constructed building within the same institutional setting, the study provides a relatively consistent overall architectural and organizational context. Nevertheless, clinic-specific characteristics, including team composition, professional structure, workflows, and organizational conditions, may differ between the participating clinics and cannot be fully separated from office type. These factors will therefore be considered as contextual characteristics and, where feasible given the sample size, as covariates in the exploratory analyses. The observational design and absence of random allocation therefore preclude causal conclusions regarding the effects of office type. Furthermore, objective architectural measures, such as acoustic measurements, spatial density, or spatial syntax metrics, were not collected; therefore, the study focuses on the implementation and use of the office concepts rather than on an objective architectural evaluation of the built environment. These limitations are addressed by integrating qualitative interview data to better understand differences in work processes and organizational context.

In addition, the generalizability of the results may be limited due to the specific setting of a single German university hospital. Furthermore, data collection takes place approximately 15 months after the relocation. This interval allowed employees and teams to adapt to the new building and office environments, thereby reducing the influence of acute relocation-related stressors. At the same time, the absence of pre-relocation baseline data means that changes associated with the relocation cannot be assessed directly. Retrospective accounts of changes following the relocation may also be subject to recall bias. The findings should therefore be interpreted as reflecting experiences of the established office environments rather than the immediate relocation process or longitudinal changes over time.

Although efforts will be made to minimize participant burden through brief diary assessments and efficient data collection procedures, fatigue and dropout cannot be completely excluded, particularly in a demanding clinical work context. Finally, voluntary participation and internal recruitment procedures may introduce self-selection bias, as employees with particularly positive or negative experiences may be more likely to participate.

Overall, by combining a conceptual framework with a mixed-methods and context-sensitive design, this study is expected to provide insights into how modern office environments are experienced by clinical staff and how spatial, organizational, and individual factors are associated with employee well-being. The findings may contribute to the development of evidence-informed recommendations for designing healthier and more effective workplace environments in healthcare settings.
